# Removal of Cr^6+^ ions and mordant violet 40 dye from liquid media using *Pterocladia capillacea* red algae derived activated carbon-iron oxides

**DOI:** 10.1038/s41598-023-45464-x

**Published:** 2023-10-25

**Authors:** Soha Mahrous Ismail Mohamed, Eda Keleş Güner, Murat Yılmaz, Ahmed El Nemr

**Affiliations:** 1 Institute of Graduate Studies and Research, Department of Environmental Studies, Alexandria University, Alexandria, Egypt; 2 Uzumlu Vocational School, Department of Property and Security, Erzincan Binali Yıldırım University, Erzincan, Turkey; 3 Bahçe Vocational School, Department of Chemistry and Chemical Processing Technologies, Osmaniye Korkut Ata University, Osmaniye, 80000 Turkey; 4 National Institute of Oceanography and Fisheries (NIOF), Kayet Bey, Elanfoushy, Alexandria, Egypt

**Keywords:** Environmental chemistry, Pollution remediation, Chemical engineering

## Abstract

In recent years, water pollution has become one of the most dangerous problems facing the world. Pollution of water with heavy metals and different dyes has caused many harmful effects on human health, living organisms and our environment. In this study, iron oxide nanomagnetic composite from *Pterocladia Capillacea* red algae-derived activated carbon (PCAC-IO) was synthesized by co-precipitation method using different iron salts and different base solutions. The synthesized nanocomposite was investigated with various characterization techniques such as FTIR, BET, SEM-EDX, TEM, XRD, and VSM. The obtained PCAC-IO adsorbent was used for Cr^6+^ ions and Mordant Violet 40 (MV40) dye removal. The adsorption mechanism of Cr^6+^ ions and MV40 dye on PCAC-IO was examined using several adsorption and kinetic isotherm models. Langmuir and Freundlich models were investigated using experimental data. Pseudo-first-order (PFO), Pseudo-second-order (PSO) and intraparticle diffusion models (IPDM) were applied to identify the adsorption mechanism. It has shown that the PSO kinetic model fits better with the experimental data obtained from PCAC-IO. This result can be interpreted as the adsorption of the adsorbate on the nanocomposite as chemical adsorption. The optimum conditions for maximum Cr^6+^ ions removal (96.88%) with PCAC-IO adsorbent occur at room temperature, 5 g L^−1^ adsorbent concentration, 100 mg L^−1^ initial pollutant concentration, pH 1 and at the end of 180 min, while maximum MV40 dye removal (99.76%), other conditions being the same, unlikely it occurred at pH 2.06 and after 45 min. The most suitable model for Cr^6+^ ions removal under the conditions of 1 L^−1^ g adsorbent concentration and 400 mg L^−1^ adsorbate concentration was Langmuir (*Q*_*max*_ = 151.52 mg g^−1^), while for MV40 removal it was Freundlich (*Q*_*max*_ = 303.03 mg g^−1^). We propose the use of activated carbon-supported iron oxide prepared from bio-waste material, especially from *Pterocladia Capillacea* red algae, as a promising adsorbent with high efficiency in the removal of Cr^6+^ ions and MV40 dye from aqueous media.

## Introduction

Organic contaminants, poisonous chemicals, synthetic compounds, and different complex molecules have recently been discovered in water supplies as a result of population development, fast industrialization and global warming. This has become an important problem for humanity. Therefore, conserving water, utilizing it wisely, and reusing it after purification can affect a variety of factors. Industrial wastewater pollutes environmental waters. These environmental waters are very dangerous because they mix with the drinking and use water of humans and animals. The way to prevent this is to purify these waters and make them reusable^1–3^.

Toxic dyes, which cause significant water pollution and environmental problems by affecting human health, aquatic life and ecological balance, are frequently used in industries such as textile, paper, chemicals, food processing, fertilizers, metal coating, batteries, pesticides, refineries, cosmetics, plastics and pharmaceuticals^1–6^. Most textile dyes are water soluble and prevent light from penetrating the aquatic environment, so they can cause harmful and toxic effects on human health, such as cancer. Azo dyes are one of the dye classes that are widely used in textile dyes and many industries^7,8^. They contain (–N=N–) bonds in their chemical structure^9^. Mordant Violet dye (C.I. 14,745) is an example of a dye containing only one azo group^10^. This dye can be found in the aquatic environment, is toxic and mutagenic to the ecosystem. It may cause harmful effects on organisms. This effect depends on the exposure time and concentration of the azo dye in water^11^.

Industrial wastewater contains heavy metals such as lead, zinc, chromium, copper, mercury, arsenic, and nickel, which are very dangerous for all living things^12–14^. Even very small amounts of these metals threaten all living things in the ecosystem. These waters, which must be given to the environment in some way, must be purified and comply with certain standards.

Chromium is one of the heavy metals whose compounds are widely used in chemical industries^15–17^. Common usage areas; leather tanning, electroplating, wood preservation, textile, metal plating, and chromate preparation industries^18,19^. Additionally, it serves as an oxidizing agent in the production of several organic compounds. The two oxidation states of chromium that are most prevalent are Cr^3+^ and Cr^6+^ ions. In an atmosphere of aerated water, all other oxidation states are unstable. It has been discovered that Cr^6+^ ion is hazardous to stem cells and living things^20^. Additionally, it is extremely mutagenic and carcinogenic, harming human health. About 100 times more hazardous than Cr^3+^ is Cr^6+^ ions^21^. Both natural and man-made sources of Cr^6+^ ion exist. Compounds containing chromium are often utilized and built up in the environment. In general, the concentration of Cr^6+^ ions in drinking water should be lower than the WHO-predicted 50 μg/L. Chromate contamination in wastewater is a problem that has to be addressed immediately. Therefore, removing Cr^6+^ ions from the environment is crucial and required^22,23^.

It is very important to remove and/or recover heavy metals and dyestuffs, which are toxic and/or carcinogenic. This can cause serious problems in terms of living and environmental health, by appropriate methods. Different methods are used to effectively remove heavy metal ions and dyestuffs from water sources. Examples of these are ion exchange, photocatalysis, coagulation, photodegradation, biological treatment, chemical oxidation/reduction, reverse osmosis and ultrafiltration^24–28^. These methods have some disadvantages such as high cost, less efficiency, other waste products and so on. On the other hand, adsorption as an alternative method has advantages such as low cost, low energy requirement, simplicity of design, ease of use, high efficiency and reuse of adsorbents^29^. It is thought that it will be a suitable choice especially when using environmentally friendly, cheap and safe adsorbents with the potential to reduce dyestuffs and heavy metals. Because of its low cost, high adsorption capacity, and simple regeneration, the use of metal-based nanoparticles is a common approach in the removal of dyes and heavy metals^30,31^.

Titanium dioxide (TiO_2_), zinc oxide (ZnO), iron oxide (Fe_3_O_4_), copper oxide (CuO), nickel oxide (NiO), and aluminum oxide (Al_2_O_3_) nanomaterials are well-known metal-based nano adsorbents for dye and heavy metal removal^32–34^. By reducing the particle size, metal oxide nanoparticles were able to absorb more substances. In order to simultaneously remove metal and organic contaminants, metal hydroxide nanoparticles are adsorbed into the skeleton of activated carbon or other porous materials^35^. By altering the pH of the fluid, it is also possible to regenerate these metal-based nanoparticles, and after numerous regenerations, they are still functional. Iron oxides are of great importance because the first magnetic material used by human beings is natural magnetite. Iron oxides are composed of different magnetic properties and different chemical components. The most important of these iron oxides are; Fe_3_O_4_ (magnetite), α-Fe_2_O_3_ (hematite), γ-Fe_2_O_3_ (maghemite), and Fe_x_O (vustite)^36^. Iron oxides have different chemical components and magnetic properties. Oxides such as Fe_3_O_4_, γ-Fe_2_O_3_, MO.Fe_2_O_3_ (M=Co, Ni, Mn etc.), which show ferrimagnetism in bulk state, shows superparamagnetism at the nanoscale^37,38^. Magnetic separation is a renewable recycling technology that is widely applied in chemistry, physics and other separation processes such as drug delivery, catalysis, magnetic resonance imaging (MRI), tissue repair and molecular diagnostics due to its fast and non-contact magnetic response^39,40^. Fe_3_O_4_ nanoparticles can adsorb various dyes from the aquatic environment. The problem with the use of magnetite as an adsorbent is its aggregation in the aqueous medium. This causes a decrease in surface area, so the adsorption capacity is reduced^6,41^. It also contains many surface functional groups involved in adsorbing these pollutants from water^42^. Due to their simple phase separation and preparation, as well 9as their ability to treat a high volume of wastewater in a short amount of time, magnetic nano adsorbents have attracted a lot of attention for the treatment of wastewater^43^.

Activated Carbon (AC), is used for different purposes in many industries. It can be defined as substances whose inner surface area and pore volume are increased by physically or chemically activating substances whose composition mostly consists of carbon^44–49^. Since activated carbon can adsorb a wide variety of molecules on its inner surface, it is used for the adsorption of various substances in the solid or gas phase^50^. Activated carbon is used in a very wide area, especially in the health sector, purification of gases and drinking water, separation of mixtures, purification in the food industry, wastewater treatment, carbon additive in the metal industry, protective clothing in the defense industry, explosives in the weapons industry, bomb-making to silence electronic systems^51,52^. A variety of raw materials, including those derived from minerals, plants, and animals, can be used to make activated carbon^53^. There are many studies on the development of activated carbon adsorbents from low-cost natural materials and some agricultural waste materials using adsorbents such as fly ash, rice paddy, corn tassel, poplar leaf powders, orange peel, nutshell, tea leaf, apricot kernel, eggshell, sugar cane pulp, coconut shell, bamboo wood sawdust and algae^54–57^. Utilizing activated carbon made from agricultural wastes has a number of benefits, including a large surface area, a microporous structure, high adsorption capacity, and high reactivity^58^. The investigations confirm that adsorbent surface area, surface shape, and pore size distribution also affect adsorption effectiveness^59^.

This study is about the preparation of activated carbon-iron oxide nanocomposites prepared from *Pterocladia Capillacea* red algae for the removal of Cr^6+^ ions and Mordant Violet 40 dye from aqueous media. No studies have been reported in the literature using iron oxide nanocomposite synthesized from *Pterocladia Capillacea* red algae for the removal of MV40 dye (C.I. 14745).

## Materials and methods

### Materials

Activated charcoal powder was purchased from Fisher Scientific, UK. *Pterocladia Capillacea* red algae were collected from Abou quir Coast, Alexandria, Egypt. Iron (III) Nitrate Nona hydrate (98%), and Iron (II) chloride hydrate were obtained from LOBA Chemie Company. Ferrous Sulphate Heptahydrate (FeSO_4_.7H_2_O), Ferric chloride (FeCl_3_), Ammonia solution (25%), Sodium hydroxide, Sodium carbonate and Ethanol were obtained from El Nasr Company. Hydrogen peroxide (50%) was purchased from Gateway Company, Hydrochloric acid solution (37%) and Sulfuric acids (98%) were purchased from Merck Company; Potassium dichromate was purchased from Sigma Aldrich Company. MV40 dye salt (C.I. 14745) was purchased from ISMA dye Company, Kafer El dwar, Egypt.

### Preparation and activation of *Pterocladia Capillacea* red algae (PC)

Several quantities of *Pterocladia Capillacea* red algae were collected from Abu Quir Coast, Alexandria, Egypt. The algae were cleaned and washed with tap water followed by distilled water to remove dirt and sand, and then dried well in an oven (105 °C). The dried algae were cut and ground by a kitchen grinder to obtain a powder form then the chemical activation for algae powder was done by pouring 600 ml of sodium carbonate solution (0.471 mol L^−1^) (10 w/w %) into 300 g from *Pterocladia Capillacea* powder and the mixture was mixed well to be homogenous, After that the mixture was dried at oven at 105 °C. After cooling at room temperature the activated algae powder was weighed. Ucar et al.^60^ used Na_2_CO_3_ solutions for activation in their research .

### Preparation of *Pterocladia Capillacea* derived activated carbon (PCAC)

A specific amount (20 g) of activated algae was taken and put in a muffle furnace device under a nitrogen gas stream at 900 °C for 1 h to form surface pores in activated carbon through its synthesis according to Ibrahim et al.^61^. The nitrogen gas was used to expel any oxygen gas present to prevent the combustion of raw algae. The temperature was raised to 900 °C within 30 min. After that, the activated carbon was cooled at room temperature, and then washed with distilled water several times and refluxed using HCl solution (1 N) for 6 h to remove any impurities or inorganic materials attached to the carbon during synthesis^62^. After that, the activated carbon was washed with hot water then followed by distilled water several times and filtered under gravity. The obtained carbon was dried in an oven (105 °C) and weighed. After that, the activated carbon was sieved using 100-micron sieve to obtain the activated carbon in powder form and called PCAC.

### Surface Modification of *Pterocladia Capillacea* derived activated carbon (PCAC after treatment or oxidation)

A specific amount (1300 ml) of hydrogen peroxide solution (12%) was poured into 60 g of activated carbon from algae (PCAC) in a 2 L glass beaker in the presence of an ozone stream for 2 h using an ozone generator. After that, the suspension was filtered and washed with distilled water several times followed by ethanol until its pH was approximately neutral. The modified activated carbon was dried in an oven at 105 °C to remove moisture and weighed to give 56.19 g of the prepared material then the material was ground well by a porcelain mortar and the obtained material was called PCAC after treatment. This method was modified according to Moreno-Castilla et al.^63^ and Valdés et al.^64^.

### Preparation of iron oxide-activated carbon nano-composite from *Pterocladia Capillacea* (PCAC-IO) by Co-precipitation method

A similar method mentioned above was used to prepare iron oxide nanocomposite from PC with some modifications. 20 g of modified activated carbon from red algae (PCAC after treatment) was dispersed in 850 ml distilled water in a 1 L round flask and then sonicated by the ultrasonic bath at 40 °C for 30 min. The mixed iron salts solutions were prepared by dissolving a 162.20 g of ferric chloride salt (0.08 mol) and 12.98 g of ferrous sulfate heptahydrate (FeSO_4_.7H_2_O) salt (0.04 mol) in 480 ml of distilled water in another flask then stirred the iron solution at 55 °C for 5 min by a sonicator, 64 ml of ammonia solution (25%) was added drop by drop into the prepared iron solutions until the pH of the iron oxide precipitate is 9.5–9.83. A flask containing the activated carbon from algae (PCAC after treatment) was poured into the prepared iron oxide contained flask in a basic medium then excess ammonia solutions (approximately 39 ml) were added until pH is 9.76. The mixture was stirred in a sonicator for 20 min at 55 °C, after this time, the flask was put on a magnetic stirrer for continuous and vigorous stirring at 90 °C for 3 h at 1300 rpm in the presence of a condenser to cool and condense the vaporized base. After that the obtained iron oxide precipitate was cooled to room temperature, filtered and washed several times with distilled water until its pH was 6 to 7, then dried at oven regulated to 105 °C to give 27.61 g of PCAC-IO powder and it was used for the removal experiments. This method was modified from Magnacca et al.^65^.

### Characterization

FT-IR Spectrophotometer with ATR unit was used to determine the surface functional group on PCAC-IO powders and PCAC after treatment. The spectrum was obtained in the wavelength range (4000–400 cm^−1^). Fourier Transform Infrared Spectrophotometer was used Bruker VERTEX 70 spectrophotometer with ATR platinum unit.

Nitrogen –adsorption Isotherm was used to measure the surface area, pore volume, and pore size distribution of the prepared samples (PCAC after treatment, PCAC-IO). Before measurements, the samples were degassed at 77 K, pressure *P*/*P*_*0*_ = 0.99. The average pore diameter and total surface area were calculated by a Brunauer, Emmett and Teller (BET) equation by BELSORP MINI II, VERSION 1.2.5 Surface area analyzer.

Our prepared samples (PCAC after treatment and PCAC-IO) were characterized by X- ray diffraction device, (model No, 202964) from Beni Sweif University to identify the phase compositions and the degree of crystallinity. The XRD pattern was obtained at Cu-Kα radiation at 10 mA with a wavelength of 1.54 Å in the 2*θ* region of 10°–80° at 25 °C.

The particle size and morphology of the PCAC-IO sample were determined using ESL Transition Electron microscopy from Scientific Researches City. The samples were prepared by using 2 mg of the powder in 5 ml ethanol and stirred in a centrifuge device. On a copper grid, a drop of the sample suspension was dropped and then the samples were tested individually.

The surface morphology, porosity of PCAC after treatment and PCAC-IO samples were investigated using an analytical Scanning Electron Microscope (JEOL JSM-6360LA). The SEM micrographs were taken at different magnifications. The powdered samples were coated with a gold layer to increase the conductivity and clear the images obtained.

VSM device from Beni Sweif University was used to measure the magnetic property of the two nano-composites PCAC-IO respectively. The magnetic field G was from + 20 KOe to − 20 KOe.

Analyticjena Spekol 1300 UV–VIS Spectrophotometer, (Model No 4560002, Cole Parmer Instrument Co., USA) was used to determine the concentration of Cr^6+^ ions and MV40 dye in aqueous solutions.

### Adsorption experiments

A stock solution of 1000 mg/L of Cr^6+^ ions solution and MV40 dye were prepared separately by dissolving a certain amount of K_2_Cr_2_O_7_ and MV40 dye salts, respectively, in 1000 ml of distilled water in a volumetric flask separately. Dilute concentrations of the Cr^6+^ ions and MV40 dye stock solutions were prepared separately from their stock solutions. The adsorption batches were done by adding different dosages of PCAC-IO Composite (100, 150, 200, 250 mg) to different concentrations of Cr^6+^ ions and MV40 dye solutions (100, 150, 200, 300, 400 ppm) separately. The volume of each concentration was 100 ml in a conical flask; the adsorbent-adsorbate suspensions were shaken at 200 rpm and room temperature using a shaker reaching 180 min of equilibrium time for each pollutant individually. A sample of each solution was taken at an interval time to analyze the concentration of residual Cr^6+^ ions and dye in solutions, respectively. The samples were analyzed until attaining equilibrium; the equilibrium was after 3 h from adsorption experiments in the case of Cr^6+^ ions and MV40 dye solutions. All experiments were doublicated and only the mean result was reported and used for the analyses.

In case of Cr^6+^ ions and MV40 dye solutions adsorption tests, 0.5 ml of Cr^6+^ ions and MV40 dye samples were taken at interval times (5, 10, 20, 30, 45, 60, 90, 120, 150, 180 min); then the composites were separated from solutions by centrifuging them at 6000 rpm for 5 min and the magnet was used to prevent the dispersion of magnetic composites in solutions and separation of the samples. After separating the solutions of Cr^6+^ ions and also the mordant dye, the filtrates were measured by the spectrophotometer device at absorbance wavelengths of 540 and 510 nm, respectively, to determine the residual concentrations of each adsorbate individually. The effect of pH, nano-composite dosage, initial concentration of adsorbates and contact time was studied in the removal experiments of Cr^6+^ ions and MV40 dye from aqueous solutions by prepared iron oxide nano-composite (PCAC-IO).

The experimental data from adsorption batches were tested by using different adsorption isotherm and kinetic models such as Langmuir, Freundlich, Pseudo-first-order, Pseudo-second-order and Intraparticle diffusion models. These models facilitated to know the mechanism of adsorption in our study on nanocomposite adsorbent. The removal % (*R*%) can be obtained by the following Eq. (1):

1 $$R\%=\frac{({C}_{0}-{C}_{t})}{{C}_{0}}\times 100$$

where *C*_0_ and *Ct* are the initial and final concentrations of adsorbate in aqueous solution respectively. Adsorption uptake or capacity *q* (mg g^−1^) can be calculated from Eq. (2):

2 $$q=\frac{\left({C}_{0}-{C}_{t}\right)\times m}{V}\times 100$$

where *m* is the mass of the iron oxide nanocomposite in grams and *V* is the volume of the adsorbate solution in Liter (L).

The pH of different solutions was studied at 1 g L^−1^ of adsorbent dosage (PCAC-IO), 100 ml solution of 100 mg L^−1^ of Cr^6+^ ions and MV40 dye concentrations individually for 180 min of contact time. The pH of Cr^6+^ ions concentrations and the MV40 dye solutions ranged from strongly acidic to strong basic solutions (pH = 1 to 11), the studied pH of the adsorbate solution was slightly decreased or increased from this pH range.

## Results and discussion

### Characterization of adsorbent

#### FTIR analyses

**Figure 1:**
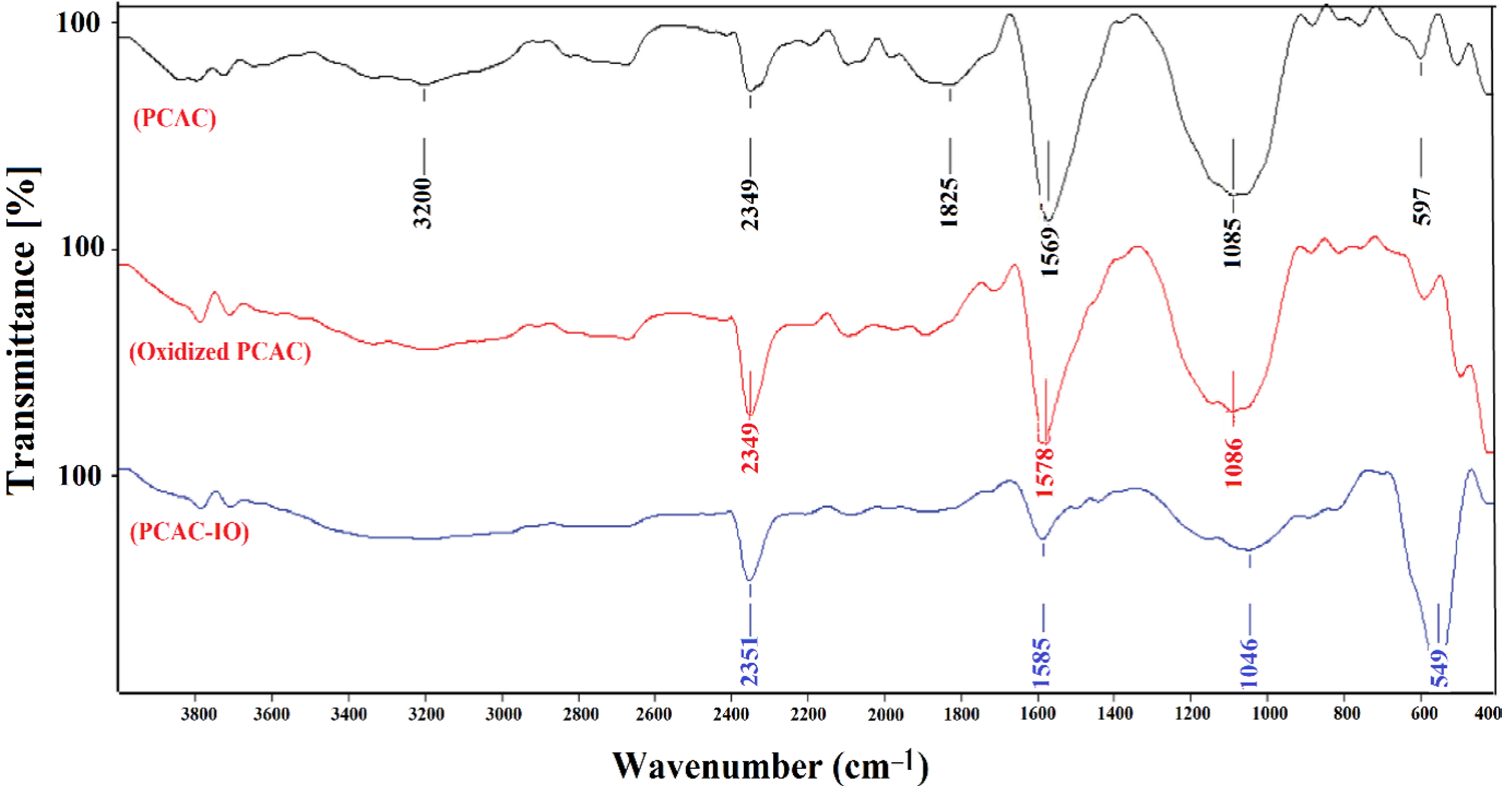
FTIR spectrum for PCAC, PCAC After oxidation and PCAC-IO Nanocomposite.

Fourier transform infrared was used to characterize the manufactured materials (PCAC, PCAC after treatment, and PCAC-IO), as seen in Fig. 1. A large peak at 3200 cm^−1^ was found in the three FTIR spectra of PCAC, PCAC after oxidation, and PCAC-IO nanocomposite; this peak is caused by the OH group. The C–C bond in the activated carbon structure with slight wave number changes were responsible for the peaks at 2394, 2349, and 2351 cm^−1^ in all spectra. The C–O deformation and C–C bond cause three peaks to appear at 1085, 1086, and 1046 cm^−1^. Due to the production of iron oxide, the Fe–O stretching bond emerged at 597 cm^−1^ with great intensity.

#### BET analyses

Using the BET equation, the surface area and pore information of the produced nano-composites and their constituent materials were calculated. The pore volume (*V*_*t*_) for the adsorbent and its precursor components was calculated using nitrogen adsorption at relative pressure *P*/*P*_*0*_ = 0.99. The nano-composite of produced activated carbon and its pore diameter were measured.

**Figure 2:**
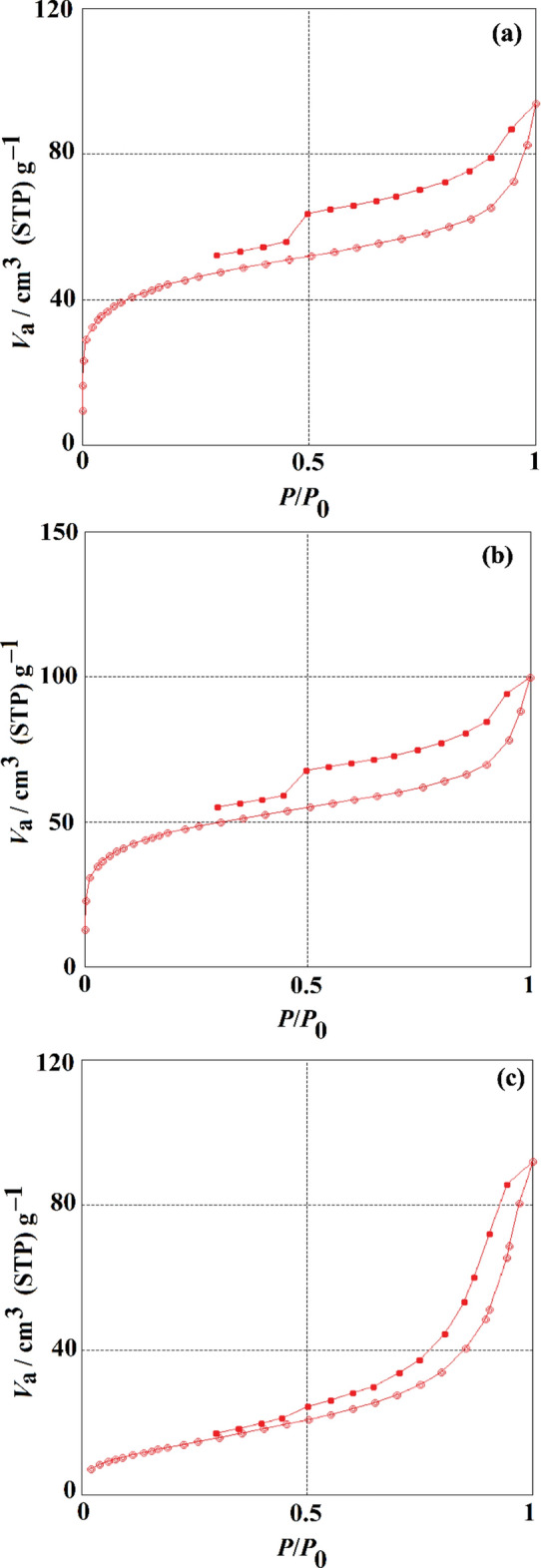
N_2_ adsorption–desorption isotherm plot of (**a**) PCAC, (**b**) PCAC after oxidation and (**c**) PCAC-IO Nanocomposite.

According to BET analysis, Table 1 and Fig. 2 demonstrate that the specific surface areas of the PCAC, PCAC after oxidation, and PCAC-IO were 164.84, 165.11, and 51.414 m^2^/g, respectively. The results demonstrate the nitrogen adsorption–desorption isotherms of the adsorbents and its precursor substances as previously mentioned; they show that they were type 4 isotherms, and the structure of PCAC before and after treatment, as well as its iron oxide nanocomposite, were mesoporous according to IUPAC classification^66^. This resulting magnetic composite (PCAC-IO) has a total pore volume of 0.136 cm^3^/g. According to the findings, the impregnation of iron oxides in a carbon structure produced a reduction in the manufactured material's surface area from 164.84 to 51.414 m^2^/g, which blocked pores in the carbon structure.

**Table 1:** Data of surface analysis of activated carbon synthesized from PC, PCAC after oxidation and its Composite (PCAC-IO).

Surface analysis	PCAC	PCAC after oxidation	PCAC-IO
Pore diameter (nm)	3.2957	3.5703	10.591
Pore volume (cm^3^ g^−1^)	0.1358	0.1474	0.1361
Surface area (m^2^ g^−1^)	164.84	165.11	51.414

#### SEM–EDX and TEM analyses

**Figure 3:**
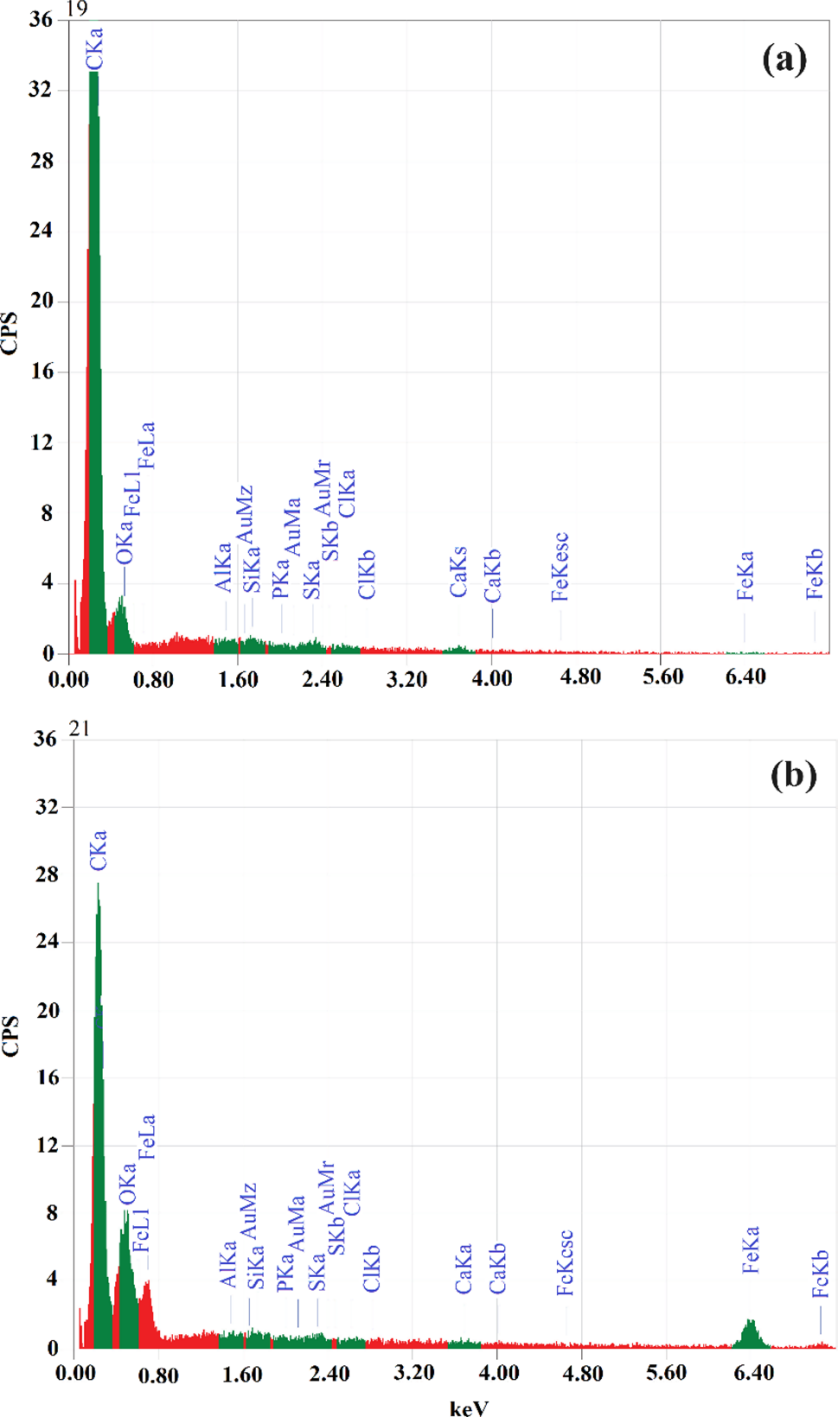
EDS data chart of (**a**) *Pterocladia capillacea* activated carbon after treatment, (**b**) PCAC-IO Nano-composite.

As seen in Fig. 3, SEM–EDX analysis was used to determine the elements of the adsorbent and iron oxide nanocomposite we prepared after processing. The PCAC-IO nano-composite and its activated carbon after oxidation PCAC were analyzed and confirmed the presence of several elements such as Carbon, Oxygen, Aluminum, Silicon, Phosphorous, Sulfur, Calcium and Chlorine with weight percentages 81.67, 14.73, 0.25, 0.64, 0.38, 0.68, 1.35, 0.30, respectively for PCAC after oxidation, the same element exists in the EDS analysis for PCAC-IO adsorbent with different weight % in addition to the presence of iron element with 28.95 weight %. Due to the presence of the element Fe in the PCAC-IO structure during synthesis, the amount of carbon in the weight reduced from 81.67 to 50.94%.

**Figure 4:**
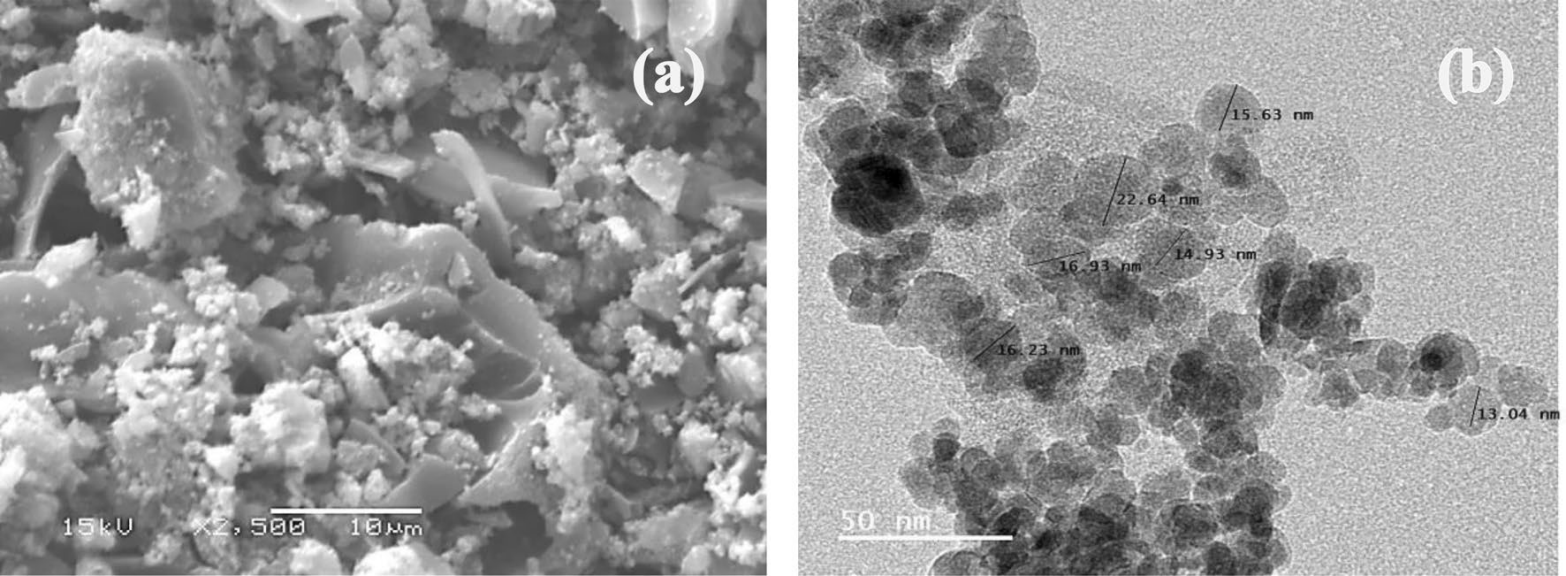
SEM (**a**) and TEM (**b**) images of PCAC-IO at 15 kV and 2500 X magnification.

As demonstrated in Fig. 4, the surface morphology and form of the produced adsorbents following treatment (oxidation) and their iron oxide nanocomposites were studied using a scanning electron microscope operating at 15 kV. Figure 4a displays a scanning electron microscopy image of a PCAC-IO nanocomposite at magnifications of 1500 × and 2,500 x. The image reveals that the adsorbent surface is rough, iron oxide nanoparticles are dispersed on the surface of the composite and block its pores, and irregularly shaped particles are also visible. These characteristics enhance the adsorption of Cr^6+^ ions and MV40 dye from its solution.

The ranges of the nano-sized composite and its form were determined using a transition electron microscope (TEM-2100), and the TEM picture of the PCAC-IO nanocomposite is displayed in Fig. 4b. The TEM image of the PCAC-IO nano-adsorbent revealed that the iron oxide (magnetite) particles were spherical and gathered together on the surface of the activated carbon, which decreased the PCAC-IO's surface area after synthesis. The particle size ranged from 13.11 to 22.64 nm, this represented the preparation of iron oxide nanocomposite.

#### VSM analyses

**Figure 5:**
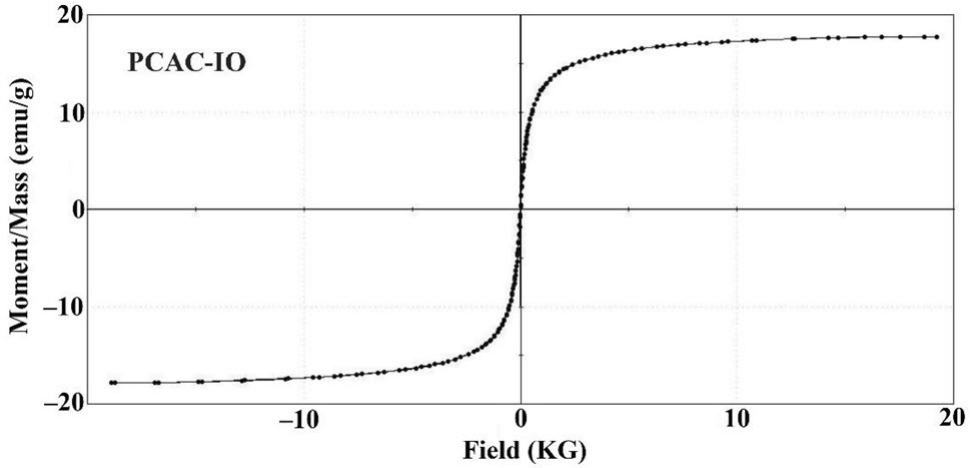
Magnetization curve for PCAC-IO nanocomposite.

In order to investigate the magnetic property, the magnetite iron oxide nano-composite (PCAC-IO) was examined. The synthesized nanocomposite magnetization curve at room temperature in a magnetic field with a cycle of − 20 to + 20 KOe. According to Fig. 5, the maximum saturation magnetization for PCAC-IO was 17.773 emu/g. It is exhausted from this result that the prepared nano-magnetic adsorbent can be separated from the water environment after the adsorption process is completed.

#### XRD analyses

**Figure 6:**
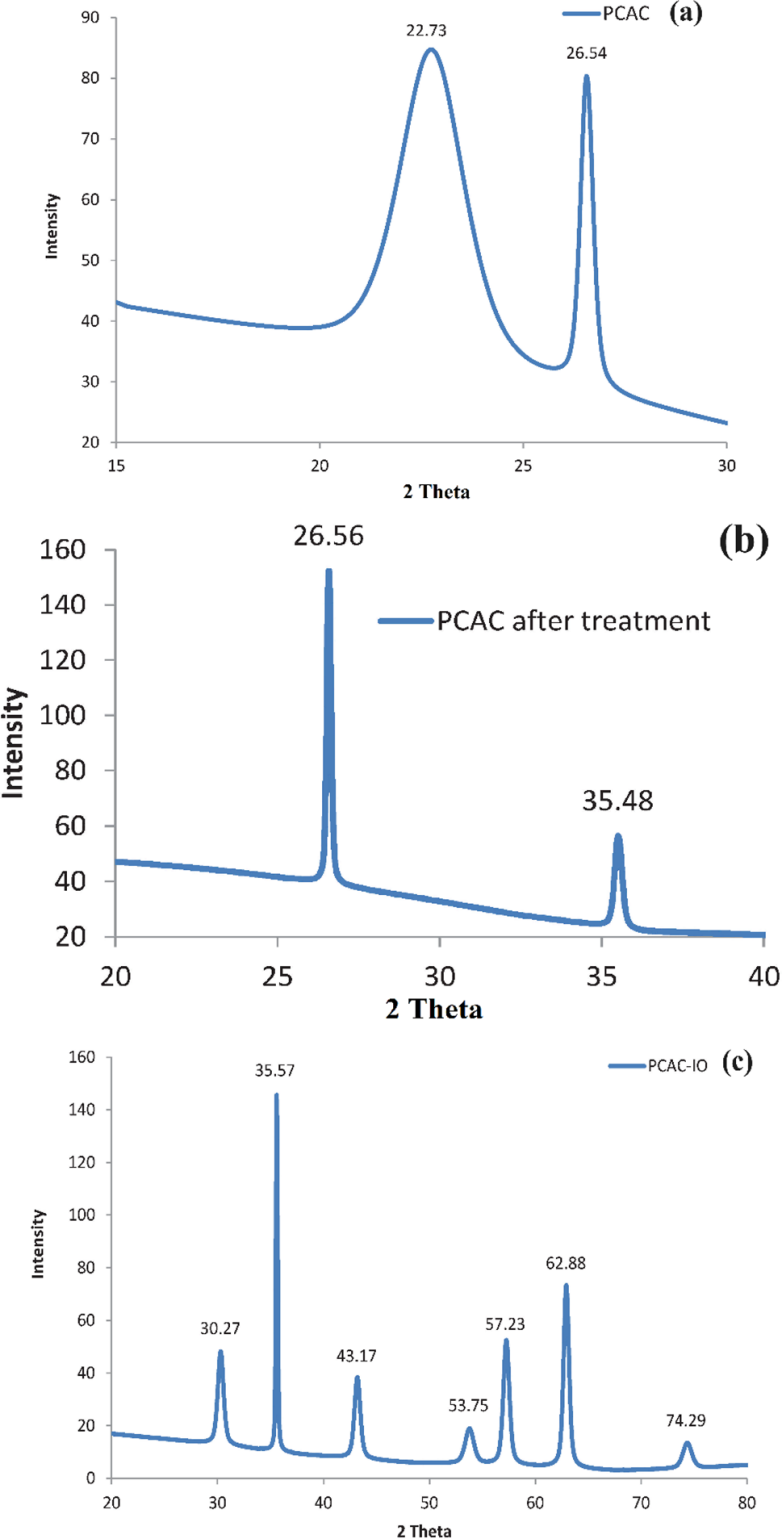
XRD graph of (**a**) PCAC (**b**) PCAC after oxidation and (**c**) PCAC/Iron oxide Nanocomposite prepared from (PCAC-IO).

The analysis of XRD of prepared nano-composites and their pristine materials at Cu- *K*α radiation at 25 °C. In the case of materials prepared from *Pterocladia capillacea* algae, Fig. 6a showed two peaks appeared at angles 2*θ* = 22.73 and 26.54 0 which are indicated the activated carbon prepared from the red algae using Na_2_CO_3_ solution as a chemical activator and a muffle furnace as a conversion device for synthesis it. The sharp peaks at 26.54 indicate the graphitic activated carbon as it was prepared at a high temperature, 900 °C. Figure 6b shows the XRD analysis of PCAC after oxidation that appears two peaks, one at 26.50 indicated to the graphitic activated carbon prepared from PC red algae and the other peak at 35.480 indicated to magnetite or maghemite with a small intensity which may be prepared before adding the Iron salts during a co-precipitation method due to the presence of impurities in the algae like Fe within its structure^67^ as previously shown in EDS analysis. Figure 6c shows the analysis of PCAC-IO nano-composite prepared from *Pterocladia capillacea* algae, the graph shows different peaks with different angle diffractions 2*θ* at 30.27, 35.57, 43.17, 53.75, 57.23, 62.88 due to the presence of iron oxide cubic structure that may be magnetite or maghemite phases^68^. The average crystalline size of the prepared iron oxide nanocomposites was determined from the XRD results; it was calculated from Scherrer’s formula,

3 $$L=\frac{K\times\uplambda }{\beta \times cos\theta }$$

where *L* is the crystalline size, *λ* is the wavelength of the X-ray, *β* is the full width of half maximum of a diffraction peak and *θ* is the angle of diffraction. *K* is Scherrer’s constant of the order of 0.89^69^. XRD results showed that the mean crystal size of PCAC-IO was 65.2 nm at 35,570 and 19.1 nm at a diffraction angle (2*θ*) of 62,880. The obtained 19.1 nm is in agreement with the results obtained from the TEM image of PCAC-IO.

### Adsorption of Cr^6+^ ions and MV40 dye on PCAC-IO

#### Effect of pH

**Figure 7:**
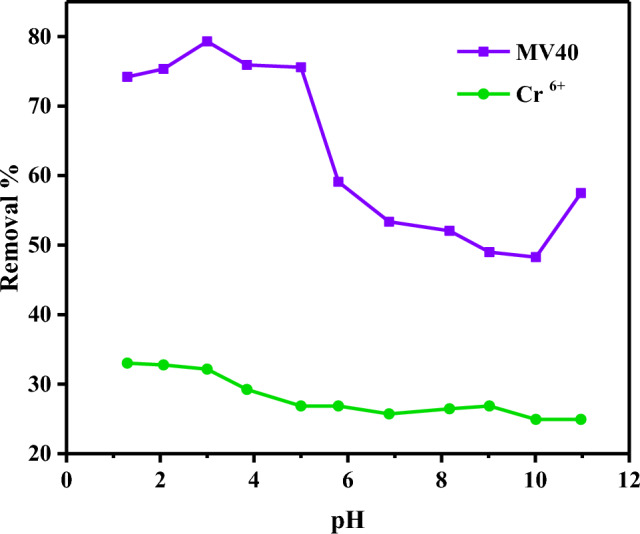
Effect of pH on the removal of Cr^6+^ ions and MV40 dye from aqueous media (Pollutant = 100 mg L^−1^, adsorbent = 1.0 g L^–1^, Time = 3 h).

The surface charge of the adsorbent is a factor that is affected by the pH of the solutions^70^. The effect of different pH solutions showed that the best removal % was in an acidic pH environment. As seen in Fig. 7, the effect of solution pH was studied by increasing the pH of Cr^6+^ ions and MV40 dye solutions from 1 to 11.6. As a result, it was observed that the removal percentage of Cr^6+^ ions decreased from 33.02% to 24.93% as the pH increased. For this reason, the optimum solution was determined as pH = 1 by obtaining the maximum removal percentage at pH 1.0.

The effect of dye solutions with different pH has been clarified that the best removal % is on acidic pH. It was observed that the MV40 dye removal increased slightly at first and then decreased continuously when the pH was increased from 1.04 to 10.48. Under optimum conditions, the best removal rate of 79.30% was obtained at pH 2.06. This result is due to the electrostatic attraction between the positively charged PCAC-IO surface and the negative charges of the MV40 dye molecules at acidic pH, but at higher pH, there is repulsion between the two opposite charges of the dye molecules and the opposite charges of the dye molecules adsorbent surface used. The same outcomes were found in El-Nemr et al.^71^.

#### Effect of PCAC-IO adsorbent dosage

**Figure 8:**
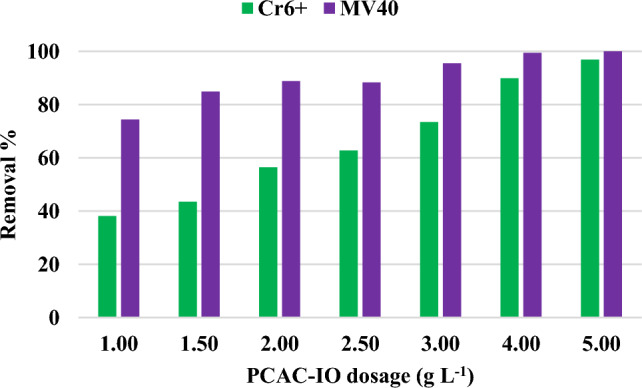
Effect of different PCAC-IO masses on the removal % of Cr^6+^ ions and MV40 dye. (Pollutant = 100 mg L^−1^, adsorbent dosage = 1.0–5.0 g L^–1^, Time = 3 h).

Different PCAC-IO nano-composite dosages (1.0, 1.5, 2.0, 2.5, 3.0, 4.0, 5.0 g L^−1^) were used to examine the mass effect of the adsorbent at initial concentrations of 100 mg L^−1^ for Cr^6+^ ions and MV40 solutions, respectively. Contact time was 3 h, and the pH of the Cr^6+^ ions solutions was 1 after the addition of the adsorbent, whereas the pH of the MV40 dye solutions was 2.06 separately. In order to determine the ultimate concentrations of the MV40 dye and Cr^6+^ ions in the solutions, solution samples were taken at regular intervals. Figure 8 shows the adsorption of Cr^6+^ ions indicating that by increasing the mass of the adsorbent (PCAC-IO) from 0.1 to 0.5 g, the removal percentage increased from 36.38 to 96.88%. The elimination percentage of MV40 was also enhanced in the adsorption trial from 74.37 to 99.76% by increasing the PCAC-IO from 0.1 to 0.5 g. In order to remove Cr^6+^ ions and dyes from their solutions at room temperature, an equilibrium duration of 3 h, and solution pH of 1 and 2,06, respectively, 0.5 g of PCAC-IO nano-composite was determined to be the optimal dosage.

#### Effect of initial adsorbates concentrations on PCAC-IO Nanocomposite

**Figure 9:**
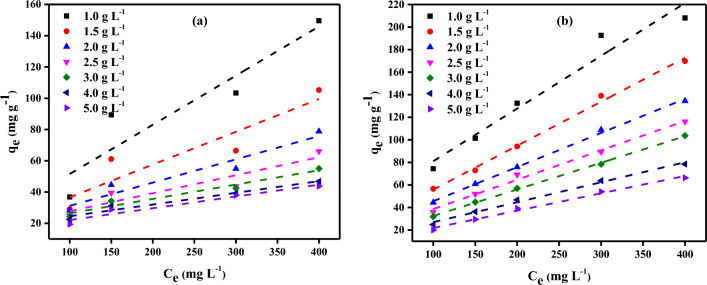
Effect of various concentrations of Cr^+6^ ions (**a**) and MV40 dye (**b**) on adsorption capacity *qe* (mg g^−1^) for each PCAC-IO concentration (pH of Cr^+6^ ions solutions = 1 and pH of MV40 dye solutions = 2.06, (Pollutant = 100–400 mg L^−1^, adsorbent dosage = 1.0–5.0 g L^–1^, Time = 3 h).

In order to examine the effect of PCAC-IO concentration, batch adsorption experiments of four different Cr^6+^ ions solution concentrations (100, 150, 300, 400 mg/L) and five MV40 dye concentrations (100, 150, 200, 300, and 400) were performed. Optimum pH values determined as pH = 1 for Cr^6+^ ions adsorption and pH = 2.06 for MV40 dye adsorption were used during the experiments. As shown in Fig. 9a, when Cr^6+^ ions were adsorbed onto PCAC-IO nano-composite, the equilibrium adsorption capacity (*q*_*e*_) increased by increasing the initial concentration of Cr^6+^ ions solutions, resulting in an adsorption capacity (*q*_*e*_) of 149.62 mg g^−1^ at fixed adsorbent dosage (PCAC-IO) = 1 g L^−1^.

As can be seen in Fig. 9b, the MV40 dye molecules were successfully adsorbed onto the PCAC-IO adsorbent. Similarly, the equilibrium adsorption capacity was increased by increasing the initial concentration of dye solutions, giving a maximum adsorption capacity of 208.08 mg g^−1^ at a fixed adsorbent dose (PCAC-IO) = 1 g L^−1^.

#### Effect of contact time using PCAC-IO

**Figure 10:**
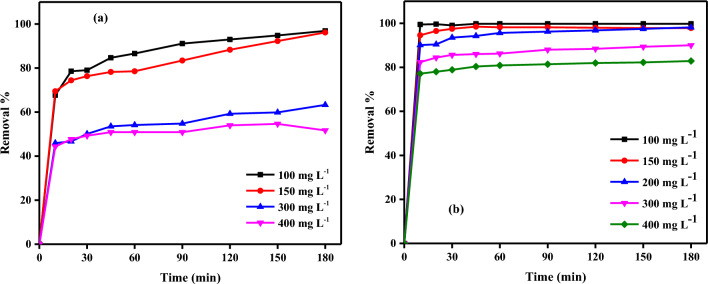
Effect of contact time on adsorption of Cr^6+^ ions (**a**) and MV40 dye (**b**) on PCAC-IO nanocomposite at optimum dose = 5.0 g L^−1^ (pH of Cr^+6^ ions solutions = 1 and pH of MV40 dye solutions = 2.06, pollutant concentration 100–400 mg L^–1^).

The effect of contact time was investigated at varying initial concentrations of Cr^6+^ ions or MV40 dye solutions, and evaluated at 5 g L^−1^ of PCAC-IO adsorbent concentration at pH of 1.0 and 2.06 of Cr^6+^ ions and dye solutions, respectively, at room temperature. According to Fig. 10a, the removal process of Cr^6+^ ions occurred very quickly. The removal of Cr^6+^ ions increased over time to reach 96.88% at equilibrium time = 180 min, which may be because there were enough unsaturated active sites on the PCAC-IO nano-composite. The removal of Cr^6+^ ions was 67.67% after only 10 min at 100 mg L^−1^ of initial concentration and 0.5 g of adsorbent dosage (PCAC-IO). When equilibrium was attained, the removal percentage of Cr^6+^ ions solutions dropped as the initial concentrations increased from 100 to 400 mg L^−1^. By raising the initial concentrations from 100 to 400 mg L^−1^ after 3 h, it reduced from 96.88 to 51.68%.

MV40 dyes were quickly removed from solutions. After only 10 min of contact time, 99.43% of the dye had been removed at an initial concentration of 100 mg L^−1^ and a PCAC-IO concentration of 5 g L^−1^; this removal percentage increased over the following 45 min to reach 99.76%. This may be because the PCAC-IO nano-composite has enough unsaturated active sites. Additionally, as previously mentioned, the clearance percentage reduced as the initial dye solution concentrations increased from 100 to 400 mg L^−1^. This occurred when the dye solution reached the equilibrium time. By increasing the initial dye concentrations from 100 up to 400 mg after 3 h, it reduced from 99.76 to 82.86% as shown in Fig. 10b.

### Adsorption ısotherm studies

As shown in Tables 2 and 3, two adsorption isotherms were examined for the adsorption of Cr^6+^ ions and MV40 dye on activated carbon-iron oxide nanocomposites made from *Pterocladia Capillacea* red algae (PC). Langmuir and Freundlich adsorption isotherm models were used. These tables display the adsorption isotherm data for the Freundlich and Langmuir models for the adsorption of MV40 dye molecules and Cr^6+^ ions from their respective aqueous environments. The characteristics of the interaction between the adsorbates and adsorbents are represented by the properties of adsorption and the parameters of each isotherm model^72^. The Langmuir model presumed that there would be a monolayer of adsorbed molecules on a uniform adsorbent surface, that there would be no interactions between the adsorbed molecules, and that the transmigration of adsorbed molecules on the adsorbent surface would not be permitted^73^. The expression for the Langmuir linear Eq. (4) is as follows:

**Table 2:** Adsorption isotherm data for Cr^6+^ ions adsorption on PCAC-IO nano-composite at room temperature.

Adsorption ısotherm model	Parameters	Activated carbon-ıron oxide composites (PCAC-IO) (g/L)						
		1.0	1.5	2.0	2.5	3.0	4.0	5.0
Langmuir	*R*^2^	0.990	0.983	0.995	0.999	0.997	0.986	0.993
	*Q*_*m*_ (mg/g)	151.52	94.34	91.74	64.94	63.69	47.85	43.48
	*K*_*a*_	0.02	0.03	0.01	0.02	0.02	0.06	0.18
Freundlich	*R*^2^	0.629	0.614	0.859	0.890	0.924	0.960	0.878
	*1/n*	0.63	0.54	0.47	0.45	0.32	0.22	0.16
	*K*_*F*_ (mg^1–1/n^L^1/n^g^−1^)	4.22	4.73	5.37	4.77	9.17	14.22	18.38

**Table 3:** Adsorption isotherm data for MV40 dye adsorption on PCAC-IO nano-composite at room temperature**.**

Adsorption ısotherm model	Parameters	Activated carbon-ıron oxide composites (PCAC-IO) (g/L)						
		1.0	1.5	2.0	2.5	3.0	4.0	5.0
Langmuir	*R*^2^	0.991	0.992	0.973	0.967	0.973	0.980	0.991
	*Q*_*m*_ (mg/g)	303.03	222.22	172.41	142.86	91.743	84.034	67.57
	*K*_*a*_	0.012	0.021	0.023	0.030	0.075	0.109	0.323
Freundlich	*R*^2^	0.990	0.973	0.982	0.993	0.974	0.998	0.990
	*1/n*	0.52	0.49	0.46	0.48	0.46	0.25	0.21
	*K*_*F*_ (mg^1–1/n^L^1/n^g^−1^)	14.12	14.32	13.84	11.89	12.32	25.21	27.73

4 $$\frac{{C}_{e}}{{q}_{e}}=\frac{1}{{K}_{a}}{Q}_{m}+\frac{1}{{Q}_{m}}\times {C}_{e}$$

where *Ce* is the concentration of adsorbate in solution (mg L^−1^) at equilibrium, *qe* is the adsorption capacity at equilibrium in mg g^−1^, *k*_*1*_ is constantly related to the free energy of adsorption (L mg^−1^), and *Q*_*m*_ is the maximum adsorption capacity at monolayer coverage in mg g^−1^. An empirical linear equation of Freundlich Isotherm assumed that the adsorbent surface was heterogeneous; the equation was expressed as shown in Eq. (5):

5 $$Ln {q}_{e }={\ln}{k}_{f}+\frac{1}{n}{\ln}{C}_{e}$$

where *k*_*f*_ (mg^1–1/n^ g^−1^ L^1/n^) and *n* are the Freundlich constants, they indicate the adsorption capacity and intensity of adsorption, respectively. The value of *1/n* in Table 2 is lower than 1, which indicated to normal Langmuir model^74^. The isotherm parameters obtained from both models due to Cr^6+^ ions adsorption on PCAC-IO were listed in Table 2. Langmuir and Freundlich's linear equations were discussed previously in Eqs. (4) and (5), respectively. The values of *1/n* in Tables 2, 3 are lower than 1, which indicated the normal Langmuir model^74^. The isotherm parameters shown in these tables indicated that the Cr^6+^ ions adsorption was fitted to the Langmuir model while MV40 dye adsorption was more fitted to the Freundlich model as shown in Fig. 11. Separation factor *R*_*L*_ was calculated by the following Eq. (6):

**Figure 11:**
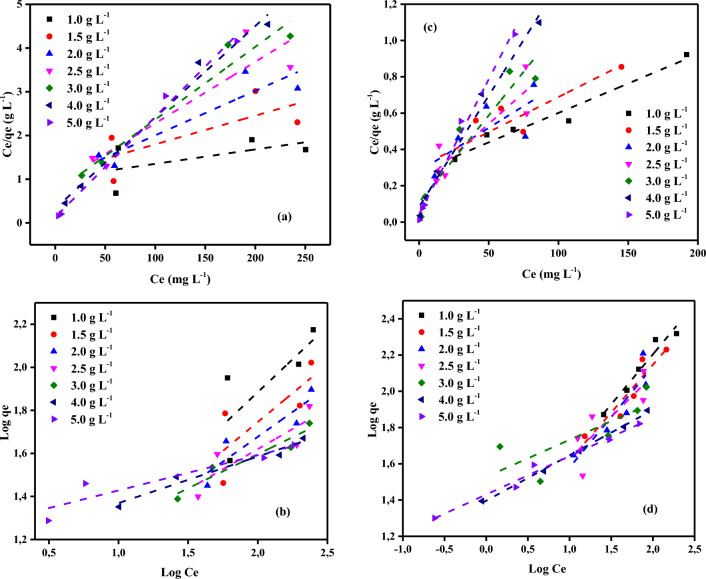
(**a**) Langmuir (**b**) Freundlich isotherms profiles for Cr^6+^ ions and (**c**) Langmuir (**d**) Freundlich isotherms profiles MV40 dye of initial concentration (100–400 mg L^−1^) on PCAC-IO doses (1.00–5.00 g L^−1^) at 25 ± 2 °C, contact time: 180 min).

6 $${\text{R}}_{L}=\frac{1}{1+{K}_{a}}{C}_{o}$$

The separation factor values determined the favorability of the adsorption process. They ranged from 0.01 to 0.45 in the case of Cr^6+^ ions adsorption and ranged from 0.01 to 0.46 where 0 < *R*_*L*_ < 1, it represented the adsorption of Cr^6+^ ions and the dye molecules on PCAC-IO nano-composite surface, respectively, was favorable. Tables 2 and 3 also showed that the maximum adsorption capacity *Q*_*m*_ from the Langmuir model was 303.03 mg g^−1^ in the case of MV40 dye adsorption at 1 g L^−1^ of PCAC-IO mass. Freundlich is more fitted due to the smallest relative error in calculations in the case of dye adsorption.

### Adsorption kinetic studies

**Figure 12:**
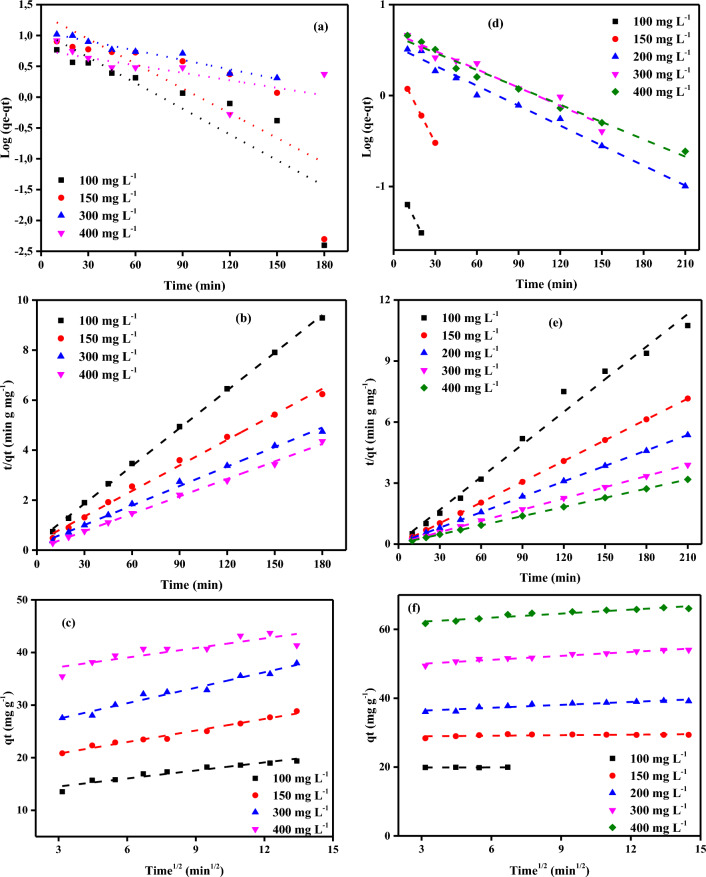
The plot of (**a**) PFO (b) PSO (**c**) IPDM of adsorption of Cr^6+^ ions and (**d**) PFO (**e**) PSO (**f**) IPDM of adsorption of MV40 dye by PCAC-IO adsorbent (Initial concentration = (100–400 mg L^−1^), Adsorbent dose = (5.0 g L^−1^), Temperature = 25 ± 2 °C).

Adsorption kinetic data were investigated by two kinetic models such as Pseudo first and Pseudo Second order models. The rate expression of Lagergren indicated pseudo-first-order^75^ as shown in Eq. (7)

7 $${\log}\left({q}_{e}-{q}_{t}\right)={\log}\left({q}_{e}\right)-\frac{{k}_{1}}{2.303}t$$

where *q*_*t*_ (mg g^−1^) is the amount of adsorbed Cr^6+^ ions on PCAC-IO adsorbent in time *t* and *k*_*1*_, (min^−1^), is the first-order rate constant, and *q*_*e*_ is the adsorption uptake at equilibrium. The straight line was obtained representing, log (*q*_*e*_ − *q*_*t*_) as the y-axis and *t* as the x-axis. *q*_*e*_ and *k*_*1*_ shown in Tables 4 and 5 were determined from the intercept and slope of the plot, respectively (Fig. 12a). The linear pseudo-second-order kinetic model was used^75^ as in Eq. (8):

**Table 4:** Comparison of the first- and second-order adsorption rate constants and calculated and experimental *qe* values for various initial Cr^6+^ ions and PCAC-IO concentrations.

Parameter			Pseudo-first-order			Pseudo-second-order			
PCAC-IO (g L^−1^)	Cr^6+^ (mg L^−1^)	*q*_*e*_ (exp.)	*q*_*e*_ (calc.)	*k*_*1*_ × 10^3^	R^2^	*q*_*e*_ (calc.)	*k*_*2*_ × 10^3^	*h*	R^2^
1.0	100	36.87	41.30	47.210	1.000	36.36	9.55	12.63	0.993
	150	89.33	18.40	2.990	1.000	78.74	5.02	31.15	1.000
	300	103.39	51.02	68.860	0.998	105.26	2.73	30.30	1.000
	400	149.62	43.32	9.670	0.891	158.73	2.06	51.81	0.994
1.5	100	29.01	11.93	5.070	0.978	27.93	14.35	11.20	1.000
	150	61.10	4.16	7.600	0.964	54.05	9.10	26.60	0.999
	300	66.45	28.62	8.290	0.983	70.42	4.73	23.47	1.000
	400	105.24	14.84	2.990	0.942	106.38	16.36	185.19	1.000
2.0	100	28.23	6.55	4.610	0.906	23.53	41.43	22.94	1.000
	150	44.57	10.92	17.730	0.992	46.51	2.42	5.24	1.000
	300	54.95	4.22	8.060	0.997	47.62	18.15	41.15	1.000
	400	78.79	4.71	7.140	0.971	78.74	6.92	42.92	0.999
2.5	100	25.10	7.71	23.490	0.908	25.97	5.22	3.52	0.996
	150	39.50	2.71	5.760	0.958	39.22	12.78	19.65	0.999
	300	43.64	5.79	2.760	0.995	45.25	2.45	5.02	0.997
	400	66.01	7.24	2.760	1.000	169.49	0.73	20.83	0.994
3.0	100	24.48	10.85	19.110	0.950	25.51	3.66	2.38	0.997
	150	34.29	3.45	15.200	0.996	34.36	13.79	16.29	1.000
	300	42.43	13.15	10.590	0.958	45.05	6.24	12.66	0.995
	400	55.01	4.78	13.590	0.961	55.25	7.22	22.03	1.000
4.0	100	22.48	8.41	14.970	0.981	23.26	4.07	2.20	0.997
	150	30.98	6.76	10.590	0.981	31.65	4.18	4.18	0.995
	300	39.12	32.22	18.650	0.964	39.68	2.66	4.19	0.994
	400	46.83	7.21	12.670	0.979	47.17	5.67	12.61	0.999
5.0	100	19.38	6.21	17.960	0.992	19.88	7.23	2.86	0.999
	150	28.85	9.38	12.210	0.944	29.33	3.58	3.08	0.995
	300	37.96	12.63	13.130	0.971	38.46	2.98	4.42	0.996
	400	43.70	10.34	28.100	0.983	44.25	5.69	11.15	0.999

**Table 5:** Comparison of the first- and second-order adsorption rate constants and calculated and experimental *q*_e_ (mg g^–1^) values for various initial MV40 dye solutions and PCAC-IO concentrations.

Parameter			Pseudo-first-order			Pseudo-second-order			
PCAC-IO (g L^−1^)	MV40 (mg L^−1^)	*q*_*e*_ (exp.)	*q*_*e*_ (calc.)	*k*_*1*_ × 10^3^	R^2^	*q*_*e*_ (calc.)	*k*_*2*_ × 10^3^	*h*	R^2^
1.0	100	74.37	23.47	31.0	0.916	74.63	8.72	48.54	1.000
	150	101.33	6.94	42.0	0.987	166.67	0.35	9.65	0.991
	200	132.42	19.43	59.0	0.927	133.33	3.41	60.61	1.000
	300	192.64	27.39	17.0	0.967	196.08	1.75	67.11	1.000
	400	208.08	81.51	14.0	0.988	212.77	0.42	19.00	0.997
1.5	100	56.53	2.90	12.0	0.972	56.50	15.21	48.54	1.000
	150	72.84	16.46	13.0	0.931	72.99	17.88	95.24	1.000
	200	94.10	5.26	45.0	0.954	95.24	7.99	72.46	1.000
	300	139.04	26.77	62.0	0.816	140.85	2.34	46.51	1.000
	400	169.87	136.30	49.0	0.914	178.57	0.63	20.20	0.997
2.0	100	44.40	3.70	13.0	0.957	44.44	14.63	28.89	1.000
	150	60.93	7.50	44.0	0.997	61.35	9.88	37.17	1.000
	200	75.87	17.37	46.0	0.891	76.34	8.50	49.50	1.000
	300	108.85	9.45	35.0	0.976	109.89	9.30	112.36	1.000
	400	134.52	120.78	94.0	0.908	138.89	2.61	50.25	1.000
2.5	100	35.08	5.23	39.0	0.975	35.97	17.56	22.73	1.000
	150	51.95	5.61	33.0	0.952	49.75	27.30	67.57	0.998
	200	69.04	7.16	16.0	0.997	67.57	15.32	69.93	0.998
	300	89.38	6.33	27.0	0.965	90.09	11.85	96.15	1.000
	400	116.12	76.03	67.0	0.926	117.65	2.74	37.88	0.999
3.0	100	31.85	2.82	95.0	1.000	32.15	66.70	68.97	1.000
	150	44.79	5.59	36.0	0.972	45.05	17.23	34.97	1.000
	200	56.97	10.38	42.0	0.979	57.14	10.49	34.25	1.000
	300	78.33	5.31	15.0	0.929	78.74	7.79	48.31	1.000
	400	103.69	18.21	16.0	0.995	104.71	2.33	25.25	0.999
4.0	100	24.78	1.23	96.0	0.965	24.81	213.70	131.58	1.000
	150	36.27	4.76	39.0	0.969	36.63	11.45	15.36	0.998
	200	46.73	10.97	42.0	0.961	46.95	9.53	21.01	1.000
	300	63.76	6.24	19.0	0.978	64.10	8.39	34.48	1.000
	400	78.46	17.06	41.0	0.902	78.74	7.27	45.05	1.000
5.0	100	19.95	8.81	12.0	0.657	19.96	738.24	294.12	1.000
	150	29.45	1.97	69.0	0.979	29.59	83.39	72.99	1.000
	200	39.26	3.51	17.0	0.969	39.53	14.29	22.32	1.000
	300	54.00	4.91	15.0	0.965	54.35	9.59	28.33	1.000
	400	66.29	4.84	16.0	0.980	66.67	10.14	45.05	1.000

8 $$\left(\frac{t}{{q}_{t}}\right)=\frac{1}{{k}_{2}{q}_{e}^{2}}+\frac{1}{{q}_{e}}(t)$$

where *k*_*2*_ (g mg^−1^) (min^−1^) is the pseudo-second-order rate constant. From the slope of the straight line *t/qt* vs. *t* plot, as shown in Fig. 12b–e, we can obtain *q*_*e*_ while *k*_*2*_ obtained from its intercept. The kinetic parameter values due to Cr^6+^ ions and MV40 dye adsorption on PCAC-IO adsorbent are separately in Tables 4, 5, 6. It showed that the adsorption process follows the pseudo-second-order (PSO) model according to correlation coefficient *R*^2^ from 0.993 to 1.000 in the case of Cr^6+^ ions adsorption and R^2^ ranged from 0.997 to 1.000 for MV40 dye adsorption and closeness of the calculated equilibrium adsorption capacity (*q*_*e*_)_calc_ to those obtained from the experimental values (*q*_e_)_exp_. *R*^2^ values for the pseudo-first-order (PFO) model are not satisfactory so, the PSO kinetic model is the best model that explained the experimental data from Cr^6+^ ions and dye adsorption on PCAC-IO. These results were interpreted that the adsorption process was chemisorption^76^. Chemisorption is sharing or exchanging electrons between the adsorbate and the active sites on the adsorbent^76^. To interpret the diffusion mechanism, the experimental results were analyzed and fitted to the intraparticle diffusion model which is expressed by the following Eq. (9):

9 $${q}_{t}={K}_{diff}{t}^{0.5}+C$$

where *K*_*diff*_ is the intraparticle rate constant (mg g^−1^ min^0.5^) and *C* is an intercept (mg g^−1^) which indicates the boundary layer effect. The intraparticle diffusion model (IPDM) was also tested on the adsorption of the MV40 dye and Cr^6+^ ions on PCAC-IO nano-composite separately, the *q*_*t*_ vs *t*^0.5^ plot was drawn as shown in Fig. 12c and f, a line didn’t pass through the origin and the intercept *C* increased by increasing the initial concentrations of adsorbates solutions as shown Fig. 12c and f. It was seen that the intraparticle diffusion model was not the only rate-determining step as discussed before. The above results showed that the interaction mechanism presented between adsorbent and pollutant was suspect to be due to electrostatic attraction between the PCAC-IO nano-composite adsorbent and MV40 dye and Cr^6+^ ions^77–79^.

**Table 6:** IPDM results of adsorption of Cr^6+^ ions and MV40 dye by PCAC-IO adsorbent (Initial concentration = (100–400 mg L^−1^), adsorbent doses = (1.0–5.0 g L^−1^), Temp. = (25 °C)).

Parameter		Cr^6+^ ions			MV40 dye		
PCAC-IO (g L^−1^)	Pollutant (mg L^−1^)	*К*_*dif*_	*C*	*R*^2^	*К*_*dif*_	*C*	*R*^2^
1.00	100	− 0.117	36.70	0.049	0.132	69.47	0.019
	150	− 1.344	92.53	0.592	0.627	91.67	0.116
	200	–	–	–	1.582	112.52	0.647
	300	0.016	80.85	0.000	2.251	162.96	0.871
	400	0.986	132.83	0.062	6.884	116.37	0.975
1.50	100	0.334	23.88	0.921	0.239	53.17	0.963
	150	− 0.700	62.32	0.467	0.142	68.52	0.023
	200	–	–	–	0.156	89.46	0.057
	300	− 0.218	59.89	0.021	2.467	113.42	0.870
	400	− 1.917	117.01	0.488	5.286	104.73	0.804
2.00	100	0.441	20.27	0.468	0.342	40.08	0.885
	150	− 0.192	45.39	0.159	0.364	53.36	0.116
	200	–	–	–	0.234	70.84	0.223
	300	0.548	41.68	0.259	0.675	101.00	0.738
	400	− 0.063	77.14	0.014	4.735	96.25	0.934
2.50	100	0.627	17.03	0.879	0.110	32.93	0.116
	150	0.228	35.82	0.840	0.659	44.93	0.446
	200	–	–	–	0.388	63.16	0.265
	300	1.526	25.22	0.589	0.424	83.95	0.741
	400	− 0.199	64.50	0.133	2.623	91.28	0.941
	100	0.778	14.41	0.994	0.037	31.20	0.162
	150	0.263	30.71	0.931	0.412	40.12	0.412
3.00	200	–	–	–	0.401	51.76	0.960
	300	0.254	36.38	0.152	0.467	71.86	0.853
	400	0.418	49.20	0.854	1.576	83.51	0.952
4.00	100	0.724	13.02	0.970	0.022	24.43	0.352
	150	0.600	22.62	0.907	0.207	33.68	0.849
	200	–	–	–	0.338	41.82	0.912
	300	1.103	23.23	0.930	0.495	57.44	0.919
	400	0.599	38.679	0.996	0.587	71.06	0.971
5.00	100	0.606	35.399	0.744	0.008	19.85	0.038
	150	0.981	24.466	0.976	0.052	28.78	0.349
	200	–	–	–	0.283	35.52	0.894
	300	0.731	18.583	0.977	0.382	48.85	0.955
	400	0.508	13.004	0.923	0.391	61.047	0.918

### Comparison of results with reported literature

The efficacy of the elimination of Cr^6+^ ions and MV40 dye using various adsorbents was compared with the PCAC-IO adsorbent in the literature review. Table 7 shows a comparison of the maximum adsorption capacities (mg g^−1^) of the adsorbents used and maximum removal (%) of pollutants in this study with other results reported in the literature. Table 7 makes it evident that the PCAC-IO adsorbent removed the Cr^6+^ ions and MV40 well.

**Table 7:** A comparison of the highest pollutant removal capabilities of some adsorbents.

Name of adsorbent	Pollutant	*Q*_*m*_ (mg·g^−1^)	References
Wheat straw and *E. adenophorum*	Cr^6+^	88.57	^80^
Magnetite nanoparticles	Cr^6+^	34.9	^81^
Rice husk-derived magnetic sorbent (RHC-Mag-2)	Cr^6+^	157.7	^82^
Active carbon derived from Lantana Camara Plant	Cr^6+^	26.25	^83^
Polyaniline hexadecyltrimethylammonium bromide (PANI/HTAB)	Mordant black 11	232.00	^84^
CMC-polyaniline hydrogel	Mordant blue 9	12.20	^85^
PCAC-IO nano-composite	Cr^6+^	151.52	This study
PCAC-IO nano-composite	Mordant violet 40	303.03	This study

## Conclusion

Iron oxide nano-composites can be prepared from *pterocladia capillacea* red algae. The magnetization saturation (Ms) increased by increasing the iron oxide: carbon ratio in the prepared nano-composites. The Cr^6+^ ions removal was 96.88% at solution pH was 1.0, the adsorbent dosage was 0.5. and 100 mg/L of initial adsorbate concentrations by PCAC-IO adsorbent. The removal % of MV40 was 99.76% obtained by using PCAC-IO adsorbent at pH of dye solutions = 2.0 and adsorbent dosage 0.5 of PCAC-IO at 100 mg/L of initial adsorbate concentrations. The removal percentage of Cr^6+^ ions and MV40 dye on both adsorbents was higher in acidic solutions than in basic solutions. PCAC nano adsorbent has high magnetization saturation of 17.773 emu/g. *Q*_*max*_ of Cr^6+^ ions on PCAC-IO was 151.52 mg g^−1^ at 1 g L^−1^, while in the case of dye, was 303.03 mg g^−1^ at 1 g L^−1^ adsorbent concentration. Freundlich model was the most fitted on MV40 adsorption using PCAC-IO. Langmuir model was more fitted on Cr^6+^ ions adsorption on PCAC-IO adsorbent. PCAC-IO nano-composites can be separated from aqueous media after treatment and the adsorption process by a magnet. The prepared Iron oxide nano-composite PCAC-IO can be used for the adsorption of Cr^6+^ ions and mordant violet 40 dye from aqueous media.

## Data Availability

The data presented in this study are available on the request from the corresponding author.
